# Long Non-coding RNA Expression Profile and Functional Analysis in Children With Acute Fulminant Myocarditis

**DOI:** 10.3389/fped.2019.00283

**Published:** 2019-07-11

**Authors:** Qingqing Liu, Yaru Kong, Bo Han, Diandong Jiang, Hailin Jia, Li Zhang

**Affiliations:** Department of Pediatrics, Shandong Provincial Hospital Affiliated to Shandong University, Jinan, China

**Keywords:** acute fulminant myocarditis, long non-coding RNAs, microarray, messenger RNA, functional analysis

## Abstract

Long non-coding RNA (lncRNA) has been associated with human diseases. To study the role of lncRNA in the pathogenic mechanism of acute fulminant myocarditis (AFM), we used a microarray to analyze lncRNA and messenger RNA (mRNA) expression in leukocyte samples from AFM patients and normal children. In total, using a 2/0.5-fold change and *P* < 0.05 as the cutoff criteria, we found that 3,101 lncRNAs and 2,170 mRNAs were differentially expressed in AFM patients. Quantitative real-time polymerase chain reaction (RT-qPCR) analysis was used to verify the microarray data. Eight differentially expressed molecules were randomly selected, including 3 upregulated lncRNAs, 3 downregulated lncRNAs, and 2 upregulated mRNAs. Among them, 7 expression profiles were consistent with the microarray results. Gene Ontology enrichment and Kyoto encyclopedia of genes and genomes (KEGG) pathway analysis were used to investigate the biological functions of these genes. Establishment of a lncRNA-mRNA co-expression network and lncRNA target predication were performed to study the molecular interactions of these molecules. Our study is the first to use microarrays to examine the lncRNA and mRNA expression profiles associated with AFM, and the results indicate that the immune system plays an important role in AFM. These findings may provide a new perspective for the pathogenesis, diagnosis, and therapy of AFM.

## Introduction

Acute fulminant myocarditis (AFM) is an inflammatory process that occurs in the myocardium and causes acute-onset heart failure (1). It is a type of myocarditis that can arise quickly, progress rapidly and lead to sudden cardiac death, with mortality rates as high as 50–70% (2). At present, many studies have aimed to determine the clinical features of AFM, including the clinical presentation, diagnosis, treatment, and outcome (3–6). However, few studies have examined the pathogenic mechanism of AFM, except for myocarditis. Previous records have confirmed the relationship between myocarditis and the immune system. On the one hand, some moieties, such as Toll-like receptors (TLRs) (7), midkine (8), and STATs (9), can activate inflammatory responses to conserve the host protective system. On the other hand, when an antigen interacts with the variable region of the T cell receptor, acquired immunity will be activated (10). Data have shown that myocarditis is closely related to signaling pathways, such as NF-κB (11), AKT/caspase-3 (12), IL-1β (13), MAPK (14), and TLR-4/NF-κB p65 (15). However, little attention has been focused on the molecular mechanism of the immune system of AFM patients.

Long non-coding RNAs (lncRNAs) are defined as transcripts of more than 200 nucleotides that are not translated into proteins (16), including antisense, intronic, intergenic, pseudogene, and retrotransposon transcripts (16). LncRNAs participate in various developmental processes, acting as signals, decoys, guides, and scaffolds in epigenetic, transcriptional, or post-transcriptional regulation (17). At present, an increasing number of lncRNAs are emerging as having roles in cardiovascular diseases (18). Some lncRNAs are involved in cardiovascular system diseases, including atherosclerosis (19), heart failure (20), and arrhythmia (21). However, data related to myocarditis are scarce, and only two studies have focused on lncRNAs and cardiac inflammation (22, 23). We aim to provide further information on lncRNAs and AFM to examine the potential pathogenicity of lncRNAs in AFM patients. In addition, microarrays have been regarded as a useful tool in transcriptome gene expression profiling. They have been broadly used to investigate the pathobiology of diverse forms of diseases (24, 25).

To determine whether lncRNAs of peripheral leukocytes might correlate with AFM, we used a microarray to analyze the dysregulated profiles of lncRNAs and mRNAs in leukocytes of children with AFM and healthy children.

## Materials and Methods

### Patients and Samples

We recruited children (aged 4 months−10 years) with AFM in this study based on the following criteria (26): sudden onset of disease, obvious initial symptoms of viral infection (especially severe fatigue and poor appetite), rapidly emerging severe hemodynamic dysfunction, serious myocardial injury, and a diffuse decrease in ventricular wall motion. The exclusion criteria included coronary heart disease, viral pneumonia, sepsis myocarditis, common acute myocarditis, AFM caused by autoimmune disease, toxic drug effects, or drug allergies. The controls consisted of healthy children.

Blood samples (3 ml) were collected into EDTA anticoagulant tubes. Leukocytes were isolated within 4 h and were immediately frozen at −80°C with 1 ml of RNeasy Total RNA Isolation Kit reagent (Qiagen, GmBH, Germany). A total of 10 children from Shandong Provincial Hospital Affiliated to Shandong University (May 2018 to February 2019) were included in this study, including 5 controls and 5 patients. The clinical characteristics of patients and controls are presented in Tables 1, 2. All patients were diagnosed with AFM using cardiac magnetic resonance imaging. Three AFM samples (M1, M2, M3) and three control samples (N1, N2, N3) were used for microarray analysis.

**Table 1:** Detailed information about the patients and controls.

**Sample ID**	**Sex**	**Age (years)**	**Hs-TnT (pg/ml)**	**BNP (pg/ml)**	**LVEF (%)**	**LVEDD (cm)**
M1	Female	9	1,028	8,577	61	4.01
M2	Male	7	7,134	8,909	20	4.66↑
M3	Male	10	1,802	7,051	46	4.11
M4	Female	7	1,105	21,835	39	4.30↑
M5	Male	0.4	3,800	>35,000	34	2.69
N1	Male	10	Normal	Normal	64	3.81
N2	Female	7	Normal	Normal	64	3.55
N3	Male	8	Normal	Normal	65	3.75
N4	Female	5	Normal	Normal	64	3.40
N5	Male	6	Normal	Normal	65	3.50

*Hs-TnT, hypersensitive troponin T, normal range, 3–14 pg/ml; BNP, brain natriuretic peptide, normal range 0–450 pg/ml; LVEF, left ventricular ejection fraction, determined by echocardiography, normal value >60%; LVEDD, left ventricular end-diastolic dimension, determined by echocardiography; ↑, larger than normal*.

**Table 2:** Clinical presentations of patients.

**Sample**	**Symptoms at onset**	**ECG**	**BP (mmHg)**	**Phenotypes**	**Assisted circulation**
M1	Repeated syncope	III° AVB	92/64	Adams-Stokes syndrome	Temporary pacemaker
M2	Headache, stomachache, emesis	I° AVB	86/60	Acute cardiac insufficiency	ECMO
M3	Fever, headache, emesis, chest pain, chest distress	ST-T change	80/51	Acute heart failure	None
M4	Fever, cough, emesis, chest pain, weakness, poor general condition	III° AVB	77/53	Acute heart failure Cardiac shock	Temporary pacemaker
M5	Fever, poor appetite, poor general condition	Inverted T wave	Undetectable	Acute heart failure Cardiac shock	ECMO

*ECG, electrocardiograph; AVB, atrioventricular block; BP, blood pressure at admission; ECMO, extracorporeal membrane oxygenation*.

### RNA Extraction

Total RNA was extracted from isolated leukocytes using the RNeasy Total RNA Isolation Kit (Qiagen, GmBH, Germany) according to the manufacturer's protocols. The RNA integrity coefficient (RIN) was determined by an Agilent Bioanalyzer 2,100 (Agilent Technologies, Santa Clara, CA, US).

### Microarray Analysis

RNA samples were used to generate biotinylated cDNA targets with the Sino Human ceRNA array V3.0 (Shanghai Sinomics Corporation, Shanghai, China) (27). Upon hybridization of the biotinylated cRNA targets, an Agilent Microarray Scanner (Agilent Technologies) was used for slide scanning. Data were extracted with Feature Extraction software 10.7 (Agilent Technologies). Raw data were normalized using the Quantile algorithm in the R package “limma.” Data analysis was conducted at Sinotech Genomics Corporation according to the protocol specified by Agilent Technologies. A fold change cutoff of 2 was adopted.

### qRT-PCR Validation

Quantitative real-time polymerase chain reaction (qRT-PCR) analysis with a LightCycler 480 (Roche, Shanghai, China) system was used to verify the microarray data. Extracted RNA was subjected to cDNA synthesis with the PrimeScriptTM RT reagent Kit (Takara, Beijing, China). qRT-PCR was conducted with TB GreenTM Premix Ex TaqTM II (Takara) as directed by the manufacturer. The relative expression levels were determined by the 2^−ΔΔCt^ method. Primer sequences used in the qRT-PCR analysis of lncRNA were shown in Table 3.

**Table 3:** Primer sequences used in the qRT-PCR analysis of lncRNA.

**LncRNA/mRNA**	**Forward primer (5^**′**^-3^**′**^)**	**Reverse primer (5^**′**^-3^**′**^)**
NONHSAT253897.1	AGTCCTCTTGCCTCCACCTTC	AGTTACCACTACTCAGCGTTTT
NONHSAT256669.1	TTAATCCGCCTAACAACCTTGC	GCCCGTTCATCTTCCAGTTC
NR_126169.1	GATTGTTCTTGTCCACCTTTGTTT	CTCACAGCATCCTTGAATCCCT
NONHSAT234238.1	CTAAGTTATGTAAAGGGAGTGG	GACAGTAAAGAGGGCTAAGAG
NONHSAT177112.1	GGCTTGTTTGTGCTTCGTGTA	AAGGAGGAACTGTTGTTTCCATT
NONHSAT232454.1	GCTGGGTAGGGTGGTGAACGA	ATGGTGGCGGGAGCCTGTAAT
IL10	CCACGCTTTCTAGCTGTTGAG	CTCCGAGACACTGGAAGGTGA
SOS2	CTTAAATGCCGGTATTTGCTG	CATTGGGTTATGTAGTCTTTGT

### GO and KEGG Pathway Analysis

Gene Ontology (GO) analysis was performed to determine the biological significance of genes in unique or representative maps of differentially expressed genes (28). KEGG pathway analysis was used to predict the underlying biological functions of dysregulated lncRNAs in pathways (29). GO and KEGG pathway analysis were performed for annotation of genes as a whole network, and differentially expressed genes were analyzed using Fisher's exact test in the R package “cluster profiler.” GO categories and pathways with *P* < 0.05 in Fisher's exact test were selected.

### Co-expression Network Analysis

We predicted the co-expression relationship between lncRNAs and mRNAs according to the dynamic changes in the gene expression signal values to investigate the relationship between lncRNAs and mRNAs. Through the co-expression network, we analyzed regulatory ability of genes and determined the core regulatory genes. The co-expression network was constructed using Cytoscape.

### Cis/trans lncRNA Target Prediction

We predicted the cis and trans targets of lncRNAs. Cis target gene prediction involved identification of mRNAs genes located within 10 kb upstream or downstream of the lncRNA as the target gene of the lncRNA. Trans target gene prediction was based on the principle of complementary sequence pairing. Blast alignment was used to obtain mRNAs that were complementary with lncRNAs. Then, RNA plex software was used to calculate the thermodynamic parameters of lncRNAs that were complementary with mRNAs, and sequences with e ≤30 were selected.

### Statistical Analysis

Statistically significant differences between groups were estimated by the Mann-Whitney *U* test using SPSS 25.0; *P* < 0.05 was considered statistically significant.

## Results

### Differential Expression of lncRNAs and mRNAs

**Figure 1:**
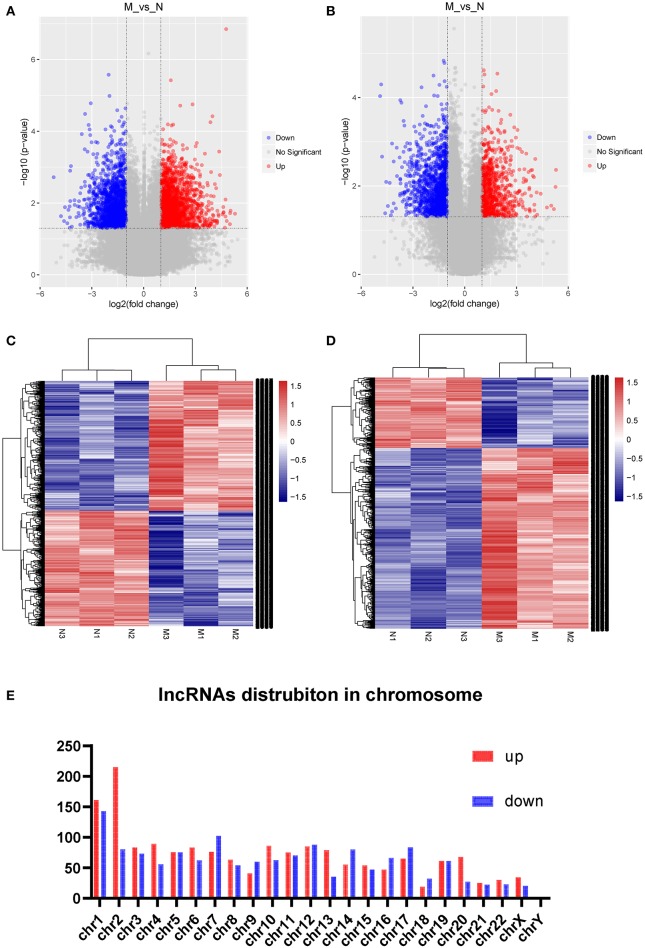
Expression profile of lncRNAs and mRNAs in AFM patients. Volcano plots were used to distinguish differentially expressed lncRNAs **(A)** and mRNAs **(B)**. Hierarchical clustering analysis of differentially expressed lncRNAs **(C)** and mRNAs **(D)**. Distribution of dysregulated lncRNAs in human chromosomes **(E)**. Red and blue represent upregulated and downregulated expression, respectively. M indicates the experimental group; N indicates the normal group.

Three samples each from the AFM patient and healthy control groups were analyzed using Sino Human ceRNA Array V3.0 microarray hybridization. Volcano plot analysis was used to assess variations in lncRNA and mRNA expression between these two populations (Figures 1A,B). Moreover, hierarchical clustering was used to distinguish AFM patients from healthy children based on gene expression data (Figures 1C,D). In total, using a 2/0.5-fold change and *P* < 0.05 as the cutoff criteria, 3,101 lncRNAs displayed differential expression in AFM patients, including 1,645 upregulated lncRNAs, and 1,456 downregulated lncRNAs. The top of 10 upregulated and downregulated lncRNAs are shown in Table 4. The distribution of differentially expressed lncRNAs on human chromosomes is shown in Figure 1E. In addition, a total of 2,170 mRNAs were dysregulated; among them, 733 were upregulated, and 1,437 were downregulated.

**Table 4:** Top 10 upregulated and downregulated (fold change ≥2 and *P* < 0.05) lncRNAs in AFM patients.

**LncRNA**	**Source**	***P***	**Fold change**	**Regulation**	**Chromosome**
ENST00000604514	Ensembl	0.047661	73.91975111	up	chr7
NONHSAT072850.2	NONCODE	0.045686	72.50661902	up	chr2
NONHSAT001832.2	NONCODE	0.002136	61.61702903	up	chr1
NONHSAT241868.1	NONCODE	0.049366	60.65420759	up	chr2
XR_001741857.1	NCBI_Gnomon	0.019768	39.22838893	up	chr4
NONHSAT067896.2	NONCODE	0.016283	32.88315135	up	chr19
XR_923024.2	NCBI_Gnomon	0.019056	32.25257773	up	chr2
NONHSAT243915.1	NONCODE	0.038709	32.01786323	up	chr20
NONHSAT167476.1	NONCODE	0.031587	28.15381126	up	chr13
NONHSAT186382.1	NONCODE	0.000000141	27.51917455	up	chr2
NONHSAT173447.1	NONCODE	0.025139761	0.017186109	down	chr16
NONHSAT251804.1	NONCODE	0.00190319	0.026931659	down	chr6
NONHSAT246340.1	NONCODE	0.023122156	0.036114184	down	chr3
NONHSAT214997.1	NONCODE	0.031748829	0.040875939	down	chr7
NONHSAT186791.1	NONCODE	0.013033559	0.042375267	down	chr2
NONHSAT162427.1	NONCODE	0.036500459	0.04659379	down	chr12
NONHSAT248478.1	NONCODE	0.015468853	0.048774319	down	chr4
NONHSAT129423.2	NONCODE	0.012387715	0.051147658	Down	chr8
NR_136191.1	NCBI_BestRefSeq	0.025003999	0.051754137	Down	chr4
NONHSAT214023.1	NONCODE	0.001251946	0.052252876	Down	chr7

### Validation by qRT-PCR

**Figure 2:**
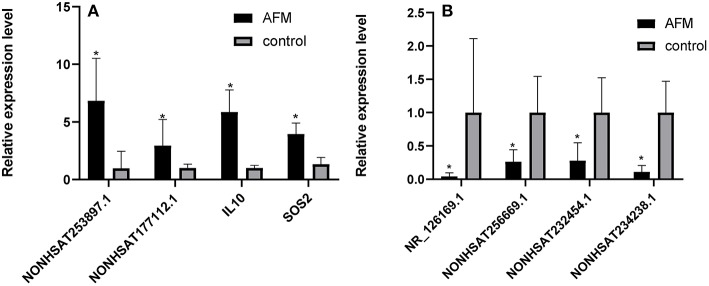
Comparison of the relative RNA expression levels between AFM patients and normal children. The expression of 2 upregulated mRNAs **(A)** and 3 downregulated and 1 upregulated lncRNA **(B)** were validated using the 2^−ΔΔCt^ method. The data are displayed as the mean ± SD, and samples of the two groups were compared using the Mann-Whitney U test. ^*^P < 0.05; AFM acute fulminant myocarditis; lncRNAs long non-coding RNAs.

To confirm the microarray data, we randomly selected 3 upregulated lncRNAs (NONHSAT253897.1, NONHSAT177112.1, and NONHSAT234238.1), 3 downregulated lncRNAs (NONHSAT256669.1, NR_126169.1, and NONHSAT232454.1), and 2 mRNAs (IL10 and SOS2), for qRT-PCR analysis (*n* = 10) (Figure 2). The results of 5 lncRNAs (NONHSAT253897.1, NONHSAT177112.1, NONHSAT256669.1, NR_126169.1, and NONHSAT232454.1) and 2 mRNAs (IL10 and SOS2) were consistent with the microarray data. The results for NONHSAT234238.1 were inconsistent with the trend sown by the microarray data.

### GO and Pathway Analysis

**Figure 3:**
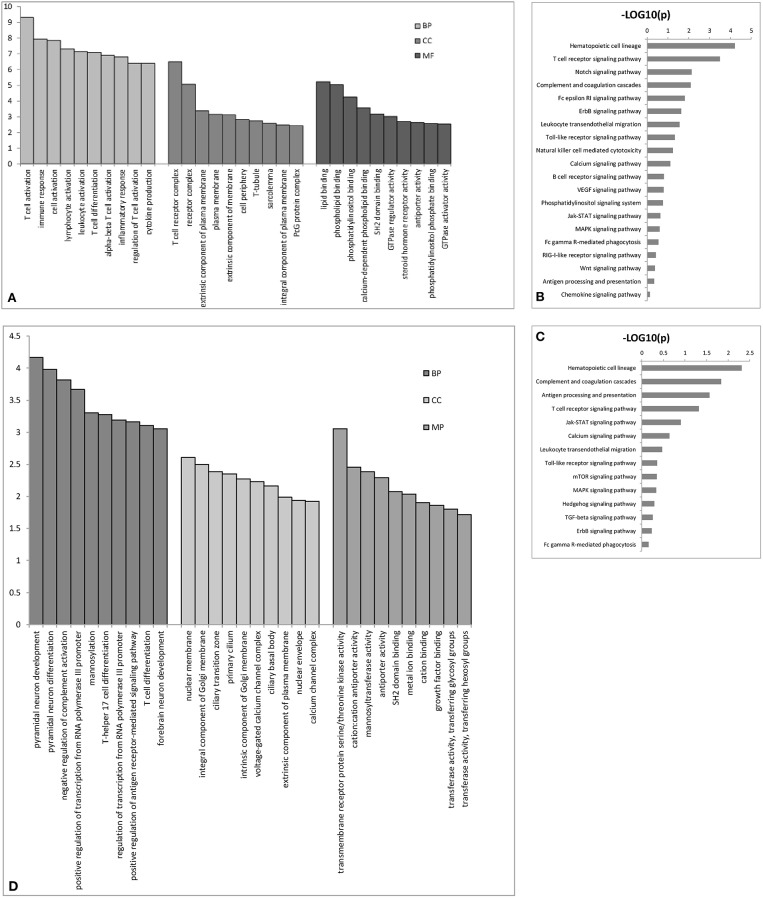
The top 30 GO terms for cis- **(A)** or trans-acting lncRNAs **(D)**. The top 10 KEGG pathways for cis- **(B)** or trans-acting lncRNAs **(C)**. BP biological process; CC cellular component; MP molecular function.

We performed GO analysis to determine the potential biological role of the dysregulated lncRNAs. The GO enrichment terms of the 30 top cis- (Figure 3A) and trans-acting lncRNAs (Figure 3D) were determined. The most prominent GO terms were T cell activation and T cell receptor complex for cis-acting lncRNAs and pyramidal neuron development, nuclear membrane, and transmembrane receptor protein serine/threonine kinase activity for trans-acting lncRNAs. According to the KEGG classification, the immune system and signal transduction were notable pathways. We chose the 10 top KEGG pathways (Figures 3C,D) within the immune system and signal transduction pathways are notable in Supplementary Figure 1; the notable pathways included hematopoietic cell lineage, complement and coagulation cascades, antigen processing, and presentation, T cell receptor signaling pathway, and Jak-STAT signaling pathway.

### lncRNA-mRNA Co-expression Network

**Figure 4:**
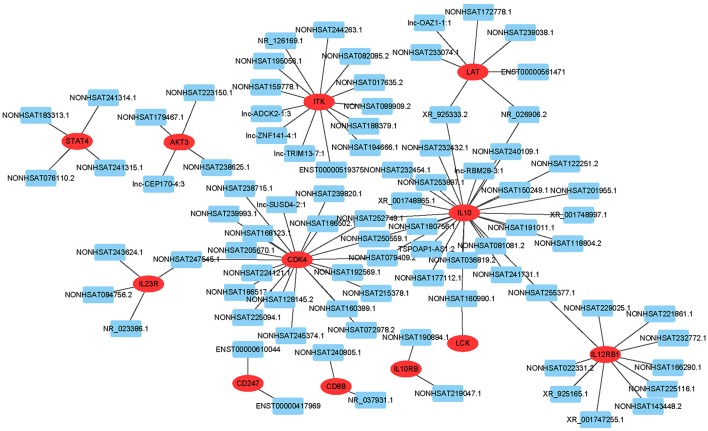
lncRNA-mRNA network analysis of the T cell receptor and Jak-STAT signaling pathways (Pearson's coefficient > 0.95). Red ovals represent mRNAs, blue rectangles represent lncRNAs, a line represents the correlation.

To predict gene function, we constructed an lncRNA-mRNA co-expression network and performed pathway analyses (Figure 4). We identified a total of 95 lncRNAs and 20 mRNAs in the T cell receptor and Jak-STAT signaling pathways (Pearson's coefficient >0.95). The co-expression network was composed of 115 network nodes and 186 connections. Each mRNA was associated with 1 to 17 lncRNAs, and each lncRNA was associated with 1–31 mRNAs.

### Cis/trans lncRNA Target Prediction

**Figure 5:**
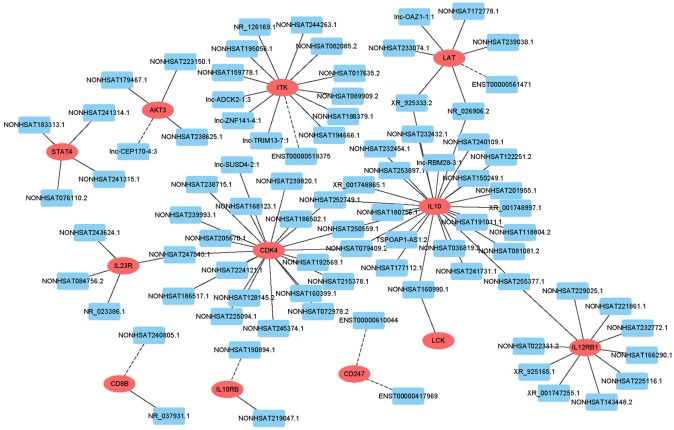
Cis/trans lncRNA target prediction in the T cell receptor and Jak-STAT signaling pathways. Red ovals represent mRNAs, blue rectangles represent lncRNAs. The solid line indicates a trans lncRNA, and the dotted line indicates a cis lncRNA.

Target prediction was performed for differentially expressed lncRNAs to investigate whether they can regulate genes and to determine the signaling pathways associated with AFM. We performed partial lncRNA target prediction in the T cell receptor and Jak-STAT signaling pathways (Figure 5). The principle of cis target gene prediction is that the function of the lncRNA is related to the protein-coding genes adjacent to its location. The basic principle of trans target gene prediction is that the lncRNA is a distant transcriptional activator or repressor of the target. The data showed that most lncRNAs acted in a cis manner. This information may aid in determining the functional mechanism of AFM.

## Discussion

AFM is characterized by acute and severe inflammation and global myocardium injury (30). The etiology and pathophysiological mechanism of AFM are unknown because of its complexity. Moreover, most of the present research focuses on the diagnosis and treatment of AFM, and the molecular mechanism of AFM is rarely reported. Many researchers believe that AFM is closely related to the immune system, which provides a platform for the study of the pathophysiological mechanisms of AFM.

LncRNAs including antisense, intronic, intergenic, pseudogene, and retrotransposon transcripts, play a vital role in biological processes (16), acting as signals, decoys, guides and scaffolds in epigenetic, transcriptional, or post-transcriptional regulation, and these molecules are emerging as dominating regulators of gene expression in the immune system. At present, most descriptions of the function of lncRNAs include modulation of the target genomic loci in a cis or in trans manner by binding to target DNA based on recognition of specific chromatin features or as an RNA-DNA heteroduplex or RNA-DNA-DNA triplex (31). LncRNAs can also function through RNA-RNA interactions. They can act as “sponges” for miRNA (32) or act by N6-methyladenosine to modify introns to form a secondary structure (33). Many studies have focused on the roles of lncRNAs in the immune system, but only a few studies have demonstrated that lncRNAs are associated with AFM. Zhang et al. (22) studied the relationship between lncRNAs and myocardial inflammation for the first time. They suggested that lncRNA TUG1 inhibits apoptosis and the inflammatory response in lipopolysaccharide (LPS)-treated H9c2 cells by downregulating mir-29b. Zhang et al. (23) demonstrated that silencing lncRNA CHRF protects H9c2 cells against LPD-induced injury via upregulation of mir-221. These two studies discussed the relationship between lncRNA and microRNA but did not examine mRNA. However, research on the transcriptional regulation of myocarditis has mainly focused on the role of mRNAs and their translated proteins.

Our study showed differential expression profiles of lncRNAs and mRNAs in children with AFM and healthy controls. We found 1,645 upregulated lncRNAs, 1,456 downregulated lncRNAs, 733 upregulated mRNAs, and 1,437 downregulated mRNAs. To verify the accuracy of the microarray, we randomly selected 8 molecules for qRT-PCR, including 3 upregulated lncRNAs (NONHSAT253897.1, NONHSAT177112.1, and NONHSAT234238.1), 3 downregulated lncRNAs (NONHSAT256669.1, NR_126169.1, and NONHSAT232454.1), and 2 upregulated mRNAs (IL-10 and SOS2). Among them, 7 molecules showed the same upregulation or downregulation trends of lncRNAs in the AFM and healthy groups. Therefore, the results from the qPCR analysis coincided with the microarray data.

Next, we used GO and KEGG analysis to determine the potential biological functions of differentially expressed lncRNAs and mRNAs. The most notable cellular processes were immune processes, including T cell activation, immune response, T cell receptor complex, negative regulation of complement activation, T-helper 17 cell differentiation, and T cell differentiation for GO terms, and those for the KEGG pathways included the complement and coagulation cascades, antigen processing and presentation, the T cell receptor signaling pathway, the Jak-STAT signaling pathway, the TLR signaling pathway, and the MAPK signaling pathway. These data further indicate the accuracy of the microarray analysis and provide more potential biological functions of lncRNAs and mRNAs related to AFM.

Furthermore, we constructed an lncRNA-mRNA co-expression network in the T cell receptor and Jak-STAT signaling pathways to obtain additional information on lncRNAs and mRNAs. We compared differentially correlated lncRNAs and mRNAs from the leukocytes of AFM patients and healthy children, which will provide a better understanding of the pathogenic mechanism of AFM.

In conclusion, the data indicate that mutual regulation between lncRNAs and mRNAs may be involved in the pathogenic process of AFM, and the results provide essential information to identify AFM.

Our study had some limitations. First, due to the low incidence of fulminant myocarditis, the sample size of the microarray analysis and that used to verify the results was small. Second, the subsequent functional verification needs to be further improved.

## Data Availability

The raw data supporting the conclusions of this manuscript will be made available by the authors, without undue reservation, to any qualified researcher.

## Ethics Statement

This study was conducted in accordance with the recommendations and guidelines of the Ethics Committee of Shandong Provincial Hospital Affiliated to Shandong University. All study participants and their parents gave written informed consent in accordance with the Declaration of Helsinki. The protocol was approved by the Ethics Committee of Shandong Provincial Hospital Affiliated to Shandong University.

## Author Contributions

QL designed the study and performed the experiments. YK, LZ, and HJ performed the experiments, analyzed the data, and wrote the manuscript. BH and DJ supervised the experiments.

### Conflict of Interest Statement

The authors declare that the research was conducted in the absence of any commercial or financial relationships that could be construed as a potential conflict of interest.

## Supplementary Material

Supplementary Figure 1 KEGG classification of lncRNAs in patients with AFM. Immune system and signal transduction are notable in the figure.
