# Supplementary figures and images for: Long Non-coding RNA Expression Profile and Functional Analysis in Children With Acute Fulminant Myocarditis
Source: Front Pediatr. 2019 Jul 11;7:283. doi: 10.3389/fped.2019.00283 (PMC6637775; doi:10.3389/fped.2019.00283)

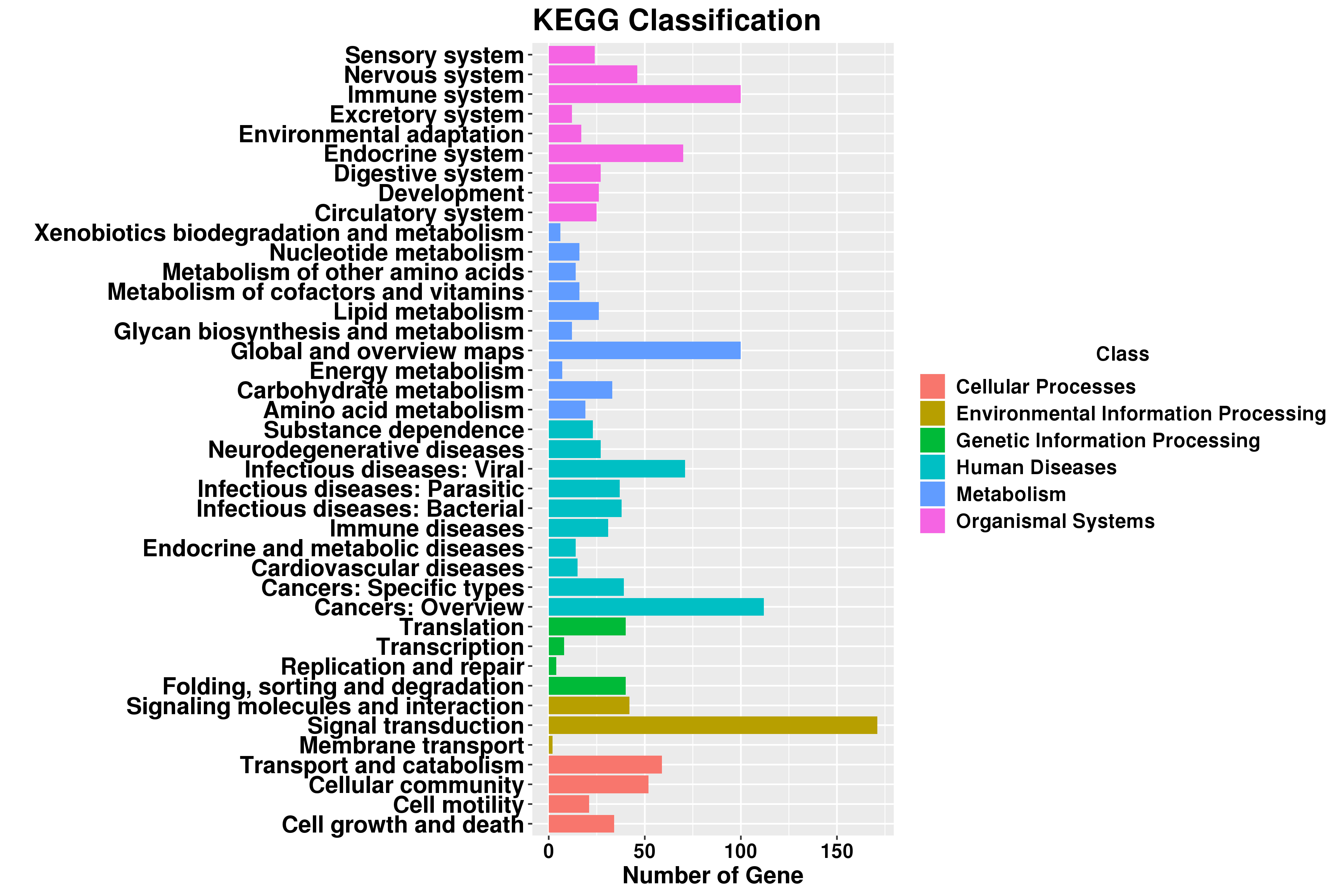

Supplement: Supplementary Figure 1 — KEGG classification of lncRNAs in patients with AFM. Immune system and signal transduction are notable in the figure. [file Image_1.PNG]
